# Re-interpreting mesenteric vascular anatomy on 3D virtual and/or physical models: positioning the middle colic artery bifurcation and its relevance to surgeons operating colon cancer

**DOI:** 10.1007/s00464-020-08242-8

**Published:** 2021-01-25

**Authors:** Bjarte T. Andersen, Bojan V. Stimec, Bjørn Edwin, Airazat M. Kazaryan, Przemyslaw J. Maziarz, Dejan Ignjatovic

**Affiliations:** 1 Department of Gastrointestinal Surgery, Østfold Hospital Trust, PO Box 300, 1714 Grålum, Norway; 2 Anatomy Sector, Teaching Unit, Faculty of Medicine, University of Geneva, Geneva, Switzerland; 3 Intervention Centre and Department of Hepatopancreatobiliary Surgery, Oslo University Hospital - Rikshospitalet, Oslo, Norway; 4 Institute for Clinical Medicine, Medical Faculty, University of Oslo, Oslo, Norway; 5 Department of Faculty Surgery, I.M. Sechenov First Moscow State Medical University, Moscow, Russia; 6 Department of Surgery N 2, Yerevan State Medical University After M.Heratsi, Yerevan, Armenia; 7 Department of Digestive Surgery, Akershus University Hospital, Lørenskog, Norway; 8 Lancet Kirurgisk Praksis, Rolvsøy, Norway

**Keywords:** Middle colic artery, Left colectomy, Colonic cancer surgery, Mesenteric vascular anatomy, Splenic flexure cancer, Accessory middle colic artery, 3D printing

## Abstract

**Background:**

The impact of the position of the middle colic artery (MCA) bifurcation and the trajectory of the accessory MCA (aMCA) on adequate lymphadenectomy when operating colon cancer have as of yet not been described and/or analysed in the literature. The aim of this study was to determine the MCA bifurcation position to anatomical landmarks and to assess the trajectory of aMCA.

**Methods:**

The colonic vascular anatomy was manually reconstructed in 3D from high-resolution CT datasets using Osirix MD and 3-matic Medical and analysed. CT datasets were exported as STL files and supplemented with 3D printed models when required.

**Results:**

Thirty-two datasets were analysed. The MCA bifurcation was left to the superior mesenteric vein (SMV) in 4 (12.1%), in front of SMV in 17 (53.1%) and right to SMV in 11 (34.4%) models. Median distances from the MCA origin to bifurcation were 3.21 (1.18–15.60) cm. A longer MCA bifurcated over or right to SMV, while a shorter bifurcated left to SMV (*r* = 0.457, *p* = 0.009). The main MCA direction was towards right in 19 (59.4%) models. When initial directions included left, the bifurcation occurred left to or anterior to SMV in all models. When the initial directions included right, the bifurcation occurred anterior or right to SMV in all models. The aMCA was found in 10 (31.3%) models, following the inferior mesenteric vein (IMV) in 5 near the lower pancreatic border. The IMV confluence was into SMV in 18 (56.3%), splenic vein in 11 (34.4%) and jejunal vein in 3 (9.4%) models.

**Conclusion:**

Awareness of the wide range of MCA bifurcation positions reported is crucial for the quality of lymphadenectomy performed. The aMCA occurs in 31.3% models and its trajectory is in proximity to the lower pancreatic border in one half of models, indicating that it needs to be considered when operating splenic flexure cancer.

**Supplementary information:**

The online version of this article (10.1007/s00464-020-08242-8) contains supplementary material, which is available to authorized users.

Since the introduction of complete mesocolic excision (CME) by Werner Hohenberger [1], the focus of the surgeon performing oncological surgery of the colon has slowly turned away from the bowel and more towards the mesentery. The appropriate terminology today deemed mesenterectomy seems to be gaining supporters within the literature [2]. Surgical procedures have also evolved from dissection within the embryological planes to a deeper understanding of the concept of “high tie”, causing another paradigm shift implying that this terminology has less to do with the blood vessel itself but much more with the fatty tissue surrounding the vessel at its origin, which is also known as the lymphovascular bundle [3–5].

When all this is taken into account a surgeon preparing to operate either right or left colectomy for cancer should be extremely interested in the position of the middle colic artery (MCA) bifurcation, since lymph node stations 222 (right) and 222 (left) according to the Japanese classification are found here [6]. Lymph node station 223 is found at the origin of the MCA, positioned on the anterior surface of the superior mesenteric artery (SMA), the superior mesenteric vein (SMV) or within the pancreatic notch. The corresponding lymph node dissection levels are D2 and D3, respectively [7]. It is of importance to mention that the common opinion of transverse mesocolon mobility is incorrect, and that in reality the transverse mesocolon is fixed in its inferior segment to the duodenum and the pancreas through the fusion fascia of Fredet, as well as along the body of the pancreas, in this manner rendering the position of the MCA bifurcation and/or the position of the MCA entry into the mesocolon fixed [8, 9].

The current surgical practice in the West is D2 lymph node dissection. Since mesenteric vascular anatomy defines lymphatic drainage [10–12], it seems that the awareness of the position of the MCA bifurcation is crucial when performing an adequate mesenterectomy. Data relating the position of the MCA bifurcation to anatomical landmarks are still missing in the literature.

The aim of this study is to determine the anatomical position of the MCA bifurcation to known anatomical landmarks (SMA/SMV) as well as evaluate its implications to the surgeon performing right or left colectomy. Additionally, the occurrence and trajectory of the accessory MCA (aMCA) and IMV is presented in the same context.

## Material and methods

The study material comprised of thirty-two anonymized high-resolution (< 1 mm slice) preoperative abdominal computed tomography (CT) datasets, acquired from patients included in the prospective multicentre trial “Safe Radical D3 Right Hemicolectomy for Cancer Through Preoperative Biphasic MDCT Angiography”. The trial has approval from the Regional Ethical Committee no: 2010/3354 and Clinical Trial Identifier NCT01351714. Patients were required to sign an informed consent form at inclusion.

All patients included had a morphologically intact left colon, including its entire vascular tree. The portovenous phase datasets underwent a detailed manual segmentation and morphometry by means of three image processing software: FDA approved Osirix MD v. 11.0.3 64-bit (Pixmeo, Bernex, Switzerland), Mimics Medical, ver. 22.0.0.524, and 3-matic Medical, ver. 14.0.0.177, (last two Windows 7 ultimate edition × 64 2017, Materialise NV, Leuven, Belgium).

### Image post‑processing

The manual editing and 3D reconstruction were carried out according to our already published protocol [4], with significant adjunct concerning the Region of Interest (ROI). Additional ROIs followed the trajectories of the MCA and MCV left branches, aMCA, if existing, and the entire suprapelvic courses of the inferior mesenteric artery (IMA) and the inferior mesenteric vein (IMV). The pixels outside ROIs were validated to air, and the ones inside the ROIs revalidated to their original value, thus erasing the surrounding tissues, and enhancing the vascular arborisation.

Datasets underwent manual thresholding (with profile line) for attributing value to voxels of the entire vascular tree. The initial mask was cropped and underwent detailed single and multiple slices editing with interpolation, following the Osirix scout files. Manual editing was facilitated by the segmentation tools: dynamic region growing, split mask, 3D LiveWire, Morphology operations, and Boolean operation tools. Finally, a 3D object mask for the vessels was calculated, without any post-editing (smoothing or triangle reduction), in order to stick as closely as possible to the original. Those masks were exported into two different file formats: the STL format (stereolithography format, commonly used in 3D printing software and hardware) and imported into the 3-matic software, as an MXP file (for virtual measuring in 3-matic). Finally, MXP files were exported as 3D PDFs with annotations as shown in 3D model 1, supplementary material.

### Virtual measurements

**Fig. 1:**
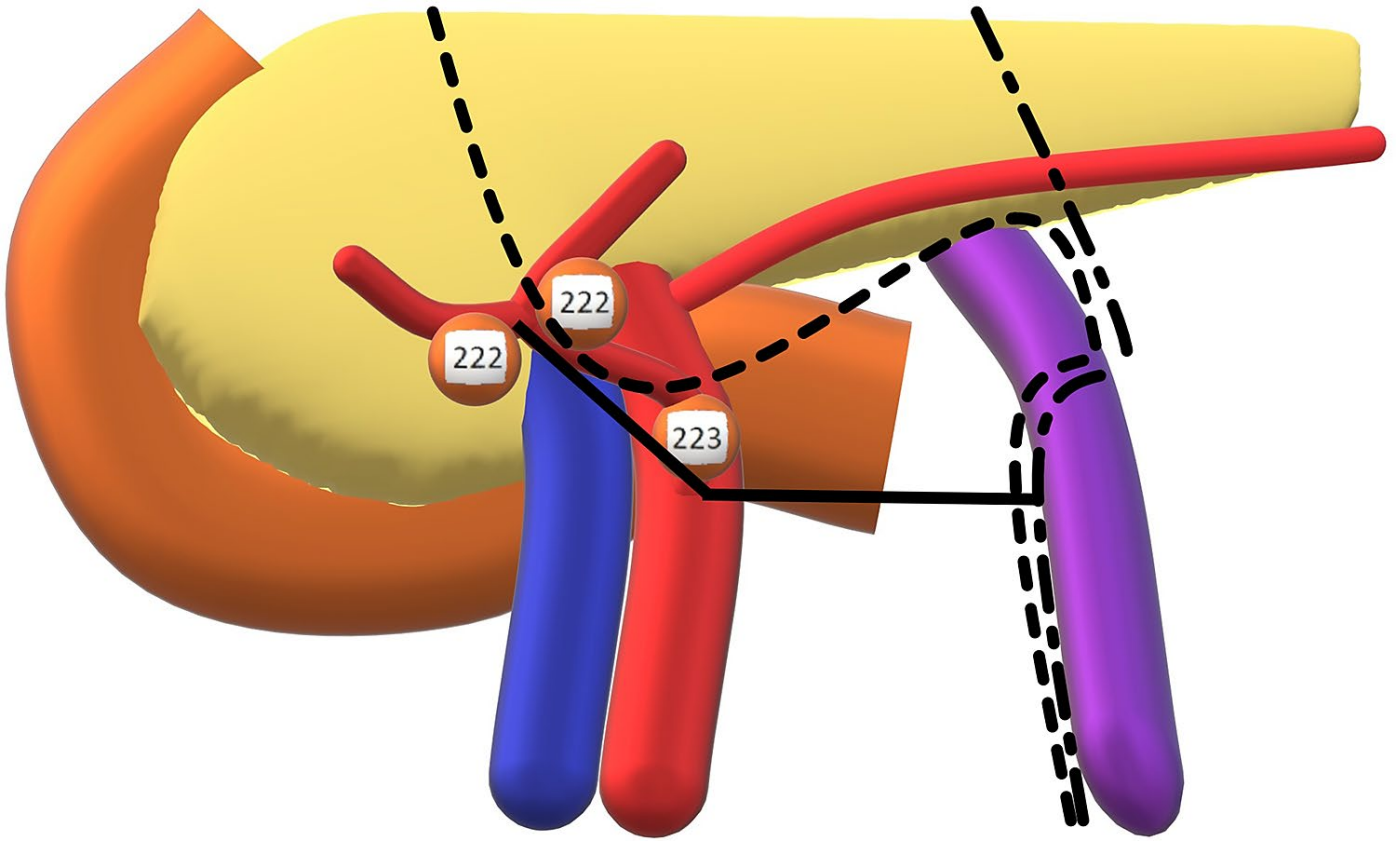
Schematic view of the middle colic artery to bifurcation with lymph nodes according to the Japanese Classification. Lymph node stations are 222 (right) following the middle colic artery (MCA) right branch, 222 (left) following the MCA left branch and 223 (central station at MCA origin). In addition, the accessory MCA is shown on the lower border of the pancreas. The solid line indicates the distance from the MCA bifurcation, via the MCA origin to the inferior mesenteric vein, the dash-dotted line indicates the standard procedure and the dotted line indicates a D2/D3 lymphadenectomy

The distances between arterial origins, the ileocolic artery (ICA), MCA and the right colic artery (RCA), as well as distance from the MCA origin to the bifurcation were measured on 3D virtual models in Osirix using length tool in a stepwise manner, except in one case where MCA originated from IMA, where Length-over-surface from the 3-matic was performed, because of a much longer course. The schematic view of MCA to bifurcation with lymph nodes according to the Japanese Classification is presented in Fig. 1.

Additional measurements include: the difference in level between the MCA origin and the level of the gastrocolic trunk of Henle (GTH) confluence, as well as the shortest horizontal distance from MCA origin to IMV. The calibres were measured at the base of the vessels, using a length tool, and measuring the largest diameter.

### 3D printing of physical models

The STL files were printed using the Form1 + (Formlabs, Sommerville, MA, USA) SLA printer. This 3D printer uses a 405 nm violet laser to harden Formlabs photopolymer photoactive resin. The printing software was PreForm version 2.20.0 supplied by Formlabs. The same setup regarding orientation, building platform and support strategies was used for all models. The resolution in the PreForm software was set to 0.05 mm, support point size was 0.60 mm with support density 1.00. After completion of the 3D print, the model was carefully placed in a container with 90% isopropanol, shaken for 2 min, and soaked for 10 min before moving it to a second container, again with 90% isopropanol, and left for another 10 min. Support structures were detached from the surface of the model by using the support removal tool supplied with the printer.

3D physical models were considered necessary and therefore created in cases of unusual or complex anatomy not readily understood on the digital models only, or when further analysis of mutual 3D relations between vessels was required to clarify the anatomy, visualizing directions, and positions of vessels.

### The vessels

The MCA bifurcation was defined as the first branching of MCA where the trajectory of both branches continues into the mesocolon. The position of the bifurcation was established on virtual and/or physical models in the anatomical coronal plane, and then classified into the following three groups:

Group I: The MCA bifurcation is positioned to the left of SMV, group II: The MCA bifurcation is positioned anterior to SMV and group III: The MCA bifurcation is positioned to the right of SMV.

The aMCA was defined as a vessel originating from SMA cranial to the “regular” MCA origin, entering the mesocolon and running towards the splenic flexure [13–16].

Virtual and/or physical models were analysed from all angles, and the two initial directions in the MCA trajectory was defined in a 3D environment (left–right; cranial–caudal; ventral–dorsal).

IMV was classified into three groups according to which vessel (SMV/splenic vein or jejunal vein) it drained into. The distances from the MCA origin to IMV were measured in a 3D environment in the transverse plane. The total distance from the MCA bifurcation to IMV was calculated by adding the two measured distances (MCA origin to the MCA bifurcation and MCA origin to IMV).

### Statistics

For continuous variables, the data are presented as median (range)/mean ± standard deviation. For categorical variables, the data are presented as number (percentage). The Shapiro–Wilk test was used to test normality in the distributions. The Kruskal–Wallis test was applied to examine differences between morphometric subgroups for non-normal distributed values and the ANOVA test for normally distributed values. Spearman's rank-order correlation test was applied to observe correlations between morphometric parameters of interest. Statistical analyses were performed using the IBM SPSS Statistics for Windows, version 25.0, IBM Corp, Armonk, NY, USA.

## Results

### Virtual and physical models

**Fig. 2:**
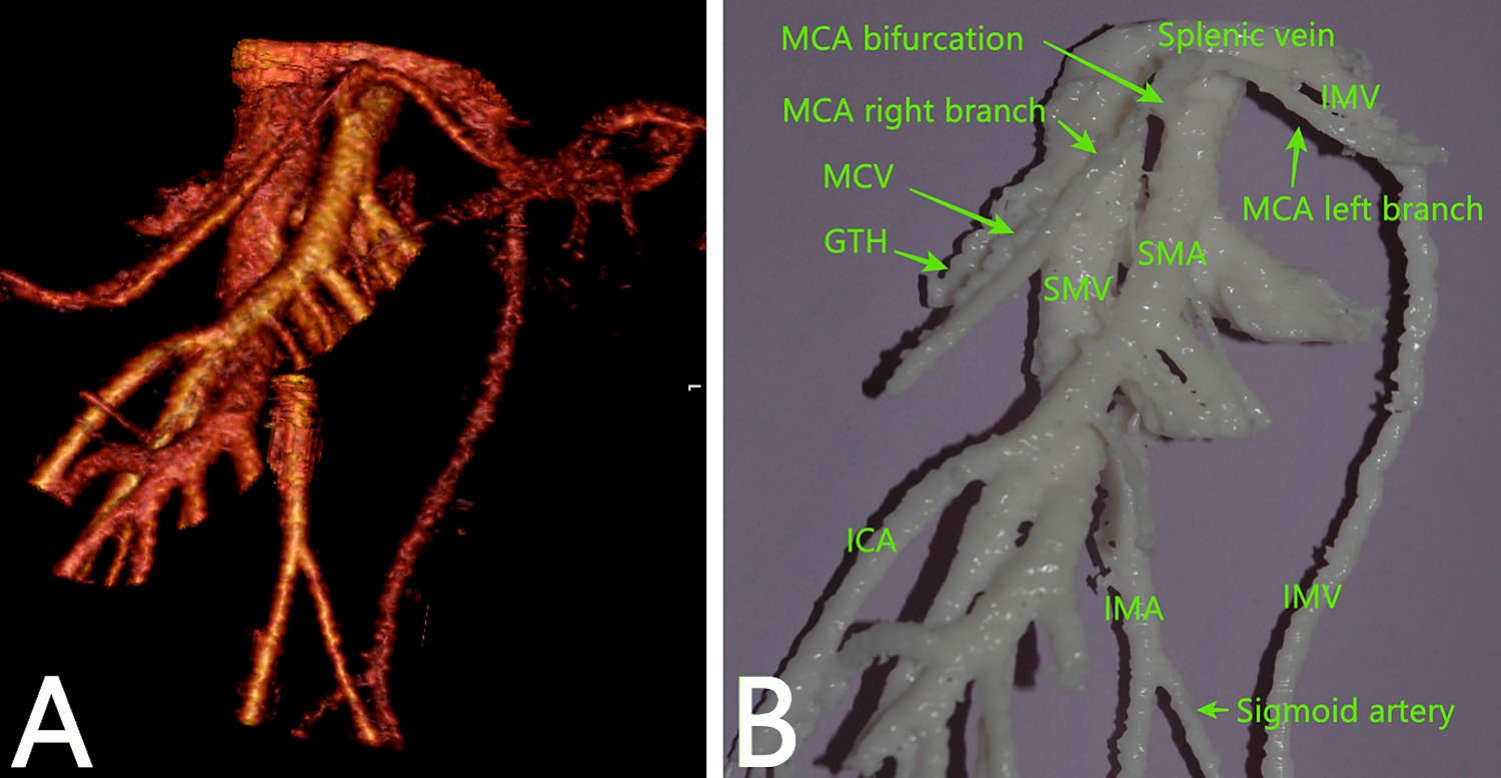
3D reconstruction of the mesenteric vascular anatomy (**A**) and 3D printed model of the same patient (**B**). *GTH* gastrocolic trunk of Henle, *IMA* inferior mesenteric artery, *IMV* inferior mesenteric vein, *ICA* ileocolic artery, *MCA* indicates the middle colic artery, MCV middle colic vein. The MCA bifurcation is positioned in front of the superior mesenteric artery (SMA). IMV opens into the splenic vein, left and distant to SMA. IMA arches downward, giving off the sigmoid artery. The sigmoid artery does not give off an ascending branch along the IMV. After a course of 5.8 cm, it gives off the left colic artery. In this patient, the accessory MCA is not present

A total of 32 3D virtual and 9 physical 3D printed models were created from 32 consecutive high-resolution CT datasets. These patients, 19 women (59.4%), mean age 67.4 ± 7.5 years were included from Nov 2nd, 2016 to June 1st, 2017 in the abovementioned clinical trial. A 3D virtual model and 3D printed model are shown in Fig. 2.

### The middle colic artery

**Fig. 3:**
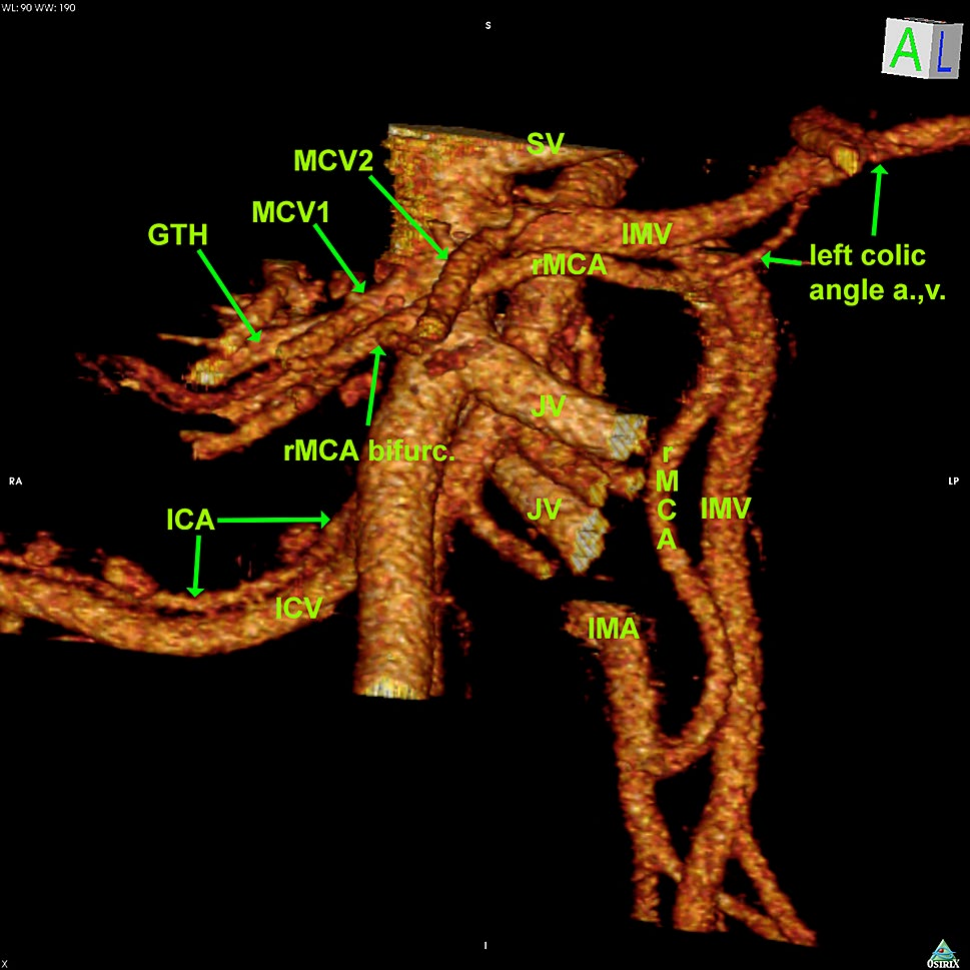
3D reconstruction of the mesenteric vascular anatomy in the patient with the replaced middle colic artery arising from the inferior mesenteric artery. *ALCA* ascending left colic artery, *GTH* gastrocolic trunk of Henle, *ICA* ileocolic artery, *ICV* ileocolic vein, *IMA* inferior mesenteric artery, *IMV* inferior mesenteric vein, *JV* jejunal vein, *MCA* middle colic artery, *MCV* middle colic vein, *rMCA* replaced middle colic artery, *SMA* superior mesenteric artery, *SMV* superior mesenteric vein, *SV* splenic vein. The MCA bifurcation is positioned right to the SMV. The IMV has a confluence into the SMV crossing the SMA anteriorly, just above the ALCA and below the SV. In this patient, the accessory MCA is not present. This anatomy is also shown in 3D virtual model 1 (Supplementary 1)

MCA was present in all patients. In one patient MCA arising from SMA could not be found and was replaced by an aberrant MCA (calibre 0.37 cm), originating from IMA. This vessel provided a branch to the splenic flexure before continuing to the MCA bifurcation that had a position anterior to SMV (length from origin to bifurcation 15.60 cm). The replaced MCA is shown in Fig. 3 and in the virtual model (Supplementary 1).

The MCA calibre at origin was 0.28 (0.18–0.40)/0.29 ± 0.06 cm. The MCA origin was found 2.37 (0.69–5.39)/2.56 ± 1.17 cm cranial to the ICA origin, and 1.18 (0.35–3.69)/1.55 ± 1.09 cm cranial to the origin of RCA when present (10 patients, 31.3%). The level of the MCA origin was found to be cranial to the level of the GTH confluence in 10 patients (31.3%), at 0.67 (0.12–1.56)/0.75 ± 0.53 cm, and caudal to the confluence of the GTH in 21 patients (65.6%) at 0.95 (0.30–2.19)/1.08 ± 0.46 cm. The length of the MCA originating from SMA from the origin to the bifurcation was 3.14 (1.18–11.52)/3.69 ± 2.15 cm, when the replaced MCA arising from IMA was included, the distance was 3.21 (1.18–15.60)/4.06 ± 2.99 cm.

**Fig. 4:**
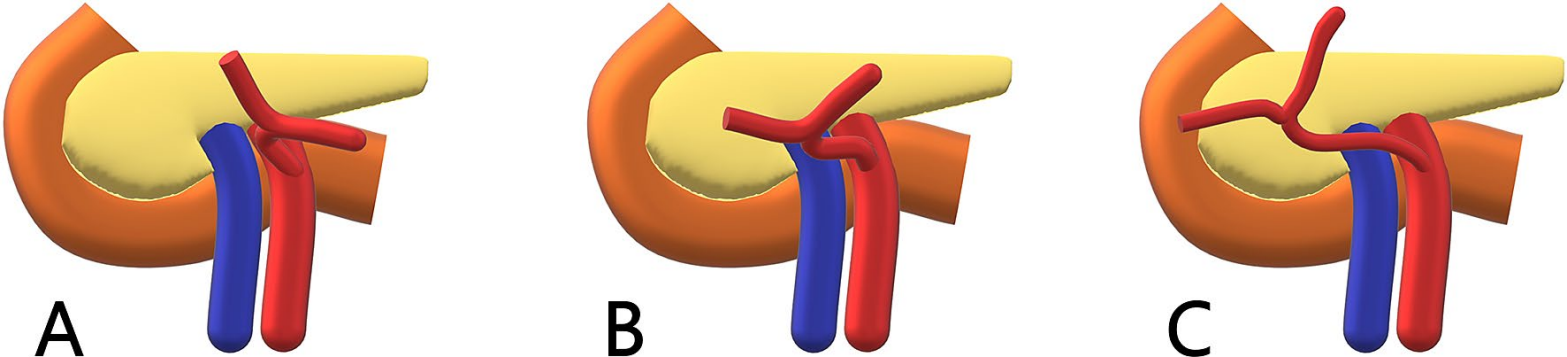
Illustration of the three positions of the middle colic artery bifurcations relative to the superior mesenteric artery, the superior mesenteric vein and the pancreas. The middle colic artery bifurcation was left to the superior mesenteric vein (SMV) in 4 cases (12.1%) (**a**), in front of SMV in 17 cases (53.1%) (**b**) and right to the SMV in 11 cases (34.4%) (**c**)

The MCA bifurcation was positioned left to SMV in 4 patients (group I, 12.1%), anterior to SMV in 17 patients, including the aberrant MCA (group II, 53.1%), and right to SMV in 11 patients (group III, 34.4%). When bifurcating right to SMV the bifurcation is found over the duodenum or the pancreatic head. The three different groups are shown in Fig. 4.

The MCA calibres had normal distribution (Shapiro–Wilk, *W* = 0.966, *p* = 0.388), while the MCA lengths to their bifurcation did not (*W* = 0.734, *p* < 0.001). The differences in the calibres between the groups were not statistically significant (the ANOVA test, *p* = 0.079). Similarly, we found significant difference in the length from the MCA origin to the bifurcation within the three groups (the Kruskal–Wallis test, *p* = 0.017).

The main direction of the MCA was found to be towards the right in 19 patients (59.4%), the most common combinations being to the right and cranial or right and ventral, both found in 6 patients (18.8%). When the initial directions were left and ventral, the bifurcation occurred left to SMV in 66.7% of the patients. When the initial directions were to the right and cranial or right and ventral, the bifurcation occurred anterior or right to the SMV in 100% of the patients. When the initial directions included left, all the MCA bifurcations were over or left to the SMV. In contrast, when the initial directions included right, all the MCA bifurcations were anterior to or right to the SMV.

In group I, the distance from the origin to the bifurcation was 1.69 (1.18–2.78)/1.83 ± 0.68 cm, group II—3.14 (1.20–15.60)/4.28 ± 3.67 and finally group III—4.19 (2.11–8.04)/4.55 ± 1.91 cm. There is a moderate, positive, monotonic correlation between the MCA length from origin to bifurcation and position of MCA bifurcation (Spearman’s rho 0.457, *n* = 32, *p* = 0.009).

In one case, an embryonal anastomotic arch between MCA (arising 2.13 cm from the MCA origin and 9.39 cm before the MCA bifurcation) and RCA was found.

### The accessory middle colic artery and vascular supply of the splenic flexure

The aMCA originating from the left-hand side of SMA was found in 10 patients (31.3%), its origin was at 2.34 (0.91–3.39)/2.21 ± 0.81 cm cranial to MCA, and the calibre was 0.19 (0.13–0.38)/0.21 ± 0.07 cm.

**Fig. 5:**
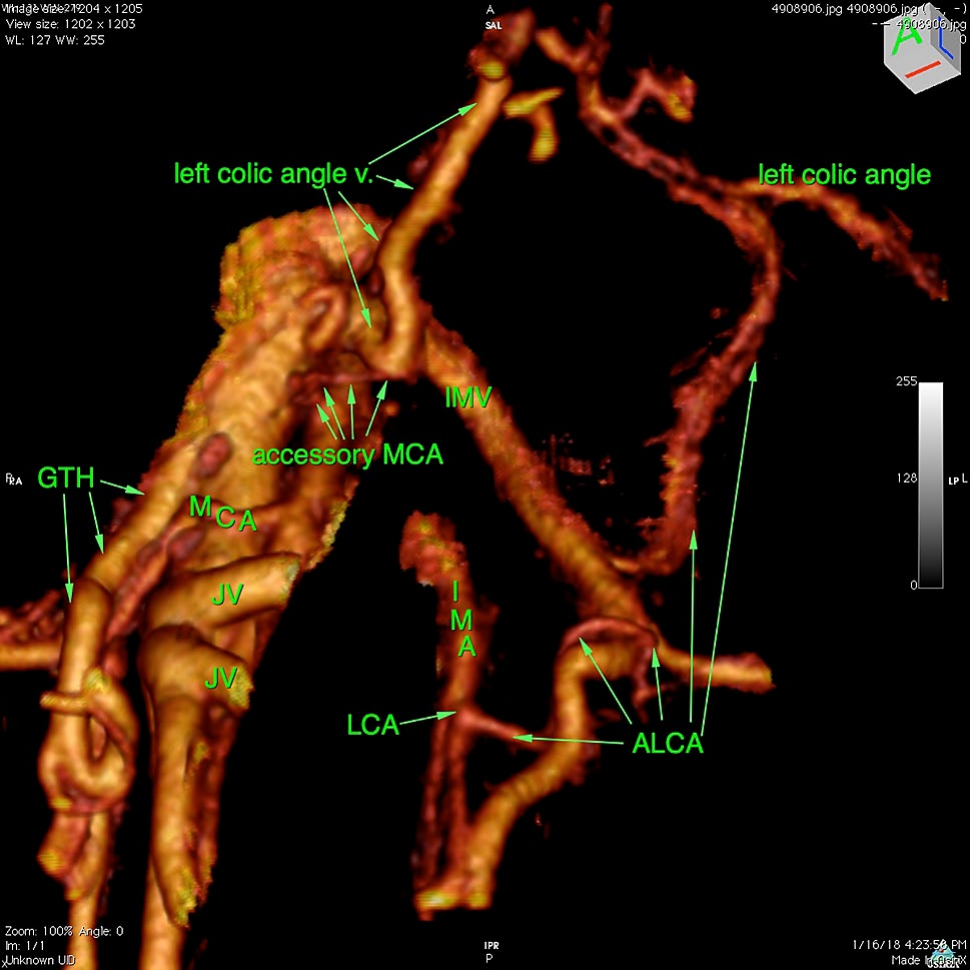
3D reconstruction of the mesenteric vascular anatomy in a patient with the accessory middle colic artery. Access. *MCA* indicates the accessory middle colic artery, *ALCA* indicates the ascending left colic artery, *GTH* indicates the gastrocolic trunk of Henle, *IMA* indicates the inferior mesenteric artery, *IMV* indicates the inferior mesenteric vein, *JV* indicates the jejunal vein, *LCA* indicates the left colic artery, *MCA* indicates the middle colic artery. The accessory MCA arises above the regular MCA origin, courses forward, then to the left, following the left colic angle vein at the lower border of pancreas. Video of this model is shown Supplementary 2.

The aMCA is directed towards the splenic flexure. The vessel will either initially follow a branch of the MCV 5 cases (50%), in the transverse mesocolon, or directly towards the flexure 5 cases (50%) in close proximity to IMV and lower border of the pancreas. This relation is shown in Fig. 5 and the video (Supplementary 2). There were no cases where the aMCA followed both an MCV branch and the IMV in the same patient.

### The inferior mesenteric vein

The horizontal distance between the MCA origin and IMV was 4.07 (1.49–5.29)/3.80 ± 0.91 cm. The distance from the MCA bifurcation through the point of the MCA origin to IMV was 7.46 (3.70–17.09)/7.86 ± 2.88 cm.

In our material, the IMV confluence was into SMV in 18 patients (56.3%), the splenic vein 11 patients (34.4%), or a terminal portion of a jejunal vein in 3 patients (9.4%). The shortest horizontal distance from the MCA origin to IMV in the subgroups opening into SMV and the splenic vein were 4.18 (1.49–4.90)/3.92 ± 3.41 and 3.95 (2.20–5.29)/3.83 ± 3.09, respectively. The shortest horizontal distance from the MCA origin to IMV in the subgroup opening into a jejunal vein was shorter 3.21 (2.03–3.51)/2.92 ± 1.48. The distances from the MCA origin to IMV had normal distribution (Shapiro–Wilk, *W* = 0.947, *p* = 0.119). The difference in distances from the MCA origin to IMV in the three groups (confluence into SMV, the splenic vein or terminal portion of a jejunal vein) was not statistically significant (the ANOVA test, *p* = 0.148).

In all cases where IMV had its confluence into the SMV, it crossed the SMA anteriorly at 2.94 (1.36–7.83)/3.07 ± 1.53 cm cranial to the MCA origin (*n* = 18), posterior to the pancreas.

## Discussion

The most important finding in this study is the wide range of possible MCA bifurcation positions, which for reasons of practicality have been classified into three groups of particular interest to the surgeon performing right or left colectomy for cancer. This find is more significant when taken in context of the transverse colon mesentery that is fixed to the duodenum, pancreatic head and notch, and not easily straightened out when right or left branches are to be divided and the corresponding lymphadenectomy performed. The fact that the MCA bifurcation can be found left to SMV, anterior to SMV or more often right to SMV is of crucial importance for the operating surgeon**.** While lengths from the MCA origin to the bifurcation have been previously reported [4, 17], the position of its bifurcation has up to date not been described in the literature.

Today, when there is little doubt that more extensive lymphadenectomy leads to improved oncological outcomes [18–22], it seems natural to consider the position of the MCA bifurcation as the key element not only while performing right colectomy, but even more so for a left-sided colonic resection [23–25]. After developing Toldt I and III plane [9, 26] underneath IMV, the crucial step in mobilizing the splenic flexure is to enter the lesser sac through the transverse mesocolon usually above the ligament of Treitz or the terminal arch of IMV [27]. If the surgeon aims towards the transverse colon, not turning towards the right of the patient, i.e. towards the MCA origin and/or the MCA bifurcation, our results clearly show that length of the remaining left branch of MCA would be at a minimum of 4.07 (1.49–5.29) cm, probably twice longer if considering the skewed trajectory of this vessel while approaching the transverse colon. With this taken into account, it becomes clear that such a lymphadenectomy for a cancer located at the splenic flexure should be deemed as an insufficient level of dissection, a D1-lymphadenectomy. Concerning our results, it seems that after developing the plane beneath IMV the interest of the surgeon should be turned towards the patients right in order to identify the MCA bifurcation/origin where the lesser sac is opened in the correct anatomical position and a D2/D3 lymphadenectomy performed [28].

On the other hand, the position of the MCA bifurcation is crucial while performing right colectomy. Several techniques have been devised in order to approach the vessels [27, 29, 30]. The transverse mesocolon needs to be mobilized through the fascia of Fredet in order to approach lymph node group 222 (right) and in this manner divide the right branch at its origin together with the lymphadenectomy. The fact that the position of the MCA bifurcation lies more often to the right does not make right colectomy simpler, since the presence of the superior right colic vein, GTH and MCV present with additional challenges.

The irrigation of the splenic flexure was first described by Griffith [31] who reported that the irrigation was from IMA in 89% of cases and SMA in 11%. Fukuoka et al. report six variants of splenic flexure irrigation, altogether 36% patients who have irrigation and drainage towards SMA [13]. Watanabe et al. [32] in a series of 31 patients reported that indocyanine real-time imaging showed that the complete or partial lymphatic drainage went towards SMA in 18 patients (58.1%). In a subgroup with 12 (38.7%) patients with a present aMCA, the irrigation always went towards SMA. CME guidelines [33] and Japanese guidelines [6] both emphasize the necessity of central ligation of the feeding artery. A feeding artery is defined in the Japanese guidelines as an artery within 10 cm of the tumour. It remains unclear if central dissection of both MCA and IMA is necessary. Watanabe et al. speculate that it is not necessary to ligate both MCA and LCA in case of splenic flexure cancer without lymph node metastasis, because in their series of 31 patients, they found no case with lymphatic drainage both towards MCA and LCA in the same patient. Since these cancers are located in a lymphovascular irrigation/drainage watershed is of importance to reconsider the question of the tumour feeding vessel of splenic flexure tumours in a dynamic fashion. Namely, and more importantly irrigation and drainage could be highly variable and interchangeable depending on the phase of the process of digestion [34–37]. Strong research evidence exists that a 30% increase in total abdominal blood flow volume occurs in response to a meal, and that this increase results in a threefold increase in the blood flow through the superior mesenteric artery. This could undoubtedly lead to shift of tumour feeding vessel since MCA is a branch of SMA, and that the IMA is not. Since all the above cited data were acquired on fasting patients (at surgery), it definitely represents only a one-sided view of the splenic flexure irrigation and requires further research. This concept indicates that a lymphadenectomy should possibly be performed around both possible feeding vessels in cases of cancer located in the splenic flexure [38].

Furthermore, our results show that aMCA is a vessel that occurs in 1 of 3 patients. The frequency of the aMCA presence reported in the literature is highly variable. It ranges from 4.1% [13] to 49.2% [16]. It seems that the reported incidence of finding this vessel has increased during the last three decades [14], reflecting the rising interest in this vessel. Our results are close to the frequency reported by Miyake et al. [14] in their series of 734 patients (36.4%), to Murono et al. [15] in their series of 205 patients (36.1%) and Tanaka et al. [39] in their series of 88 patients (30.7%).

The aMCA is directed towards and supplies the splenic flexure. We find that 50% of them follow the MCV left branch in the transverse mesocolon, and 50% are found near the IMV in close proximity to the inferior border of the pancreas. This latter position makes it difficult to find when entering the lesser sac anterior to IMV, possibly leaving an intact tumour supplying vessel in situ together with the draining lymph nodes. It also seems debatable if lymphadenectomy at the MCA bifurcation should be performed when aMCA is found. Three-dimensional reconstruction of the mesenteric vasculature could be valuable tool in decision making as it has been shown to be a valuable tool for the operating surgeon [4, 15, 40], improving the lymphadenectomy and decreasing operative time and blood loss [41]. Nevertheless, operating surgeons that do not have access to similar reconstructions can orient themselves on the anatomy through careful analysis of the preoperative CT scan. Our results might be used as an aid to localize the positions of the MCA bifurcation and aMCA. Regarding the MCA bifurcation, note that a longer MCA bifurcated over or right to SMV and a shorter bifurcated left to SMV. Further, when preoperative examinations suggest aMCA following IMV, one should anticipate a vessel in close proximity to the inferior border of the pancreas which has a high probability of being accompanied by lymphatic drainage from the splenic flexure. In addition, it is necessary to mention when the aMCA is in concern that considerable confusion can arise due the different nomenclature deployed in the literature for a vessel with a similar origin and trajectory, the superior left colic artery [42], the left accessory aberrant colic artery [32, 43], accessory left colic artery [13, 44] or most recently the transverse colon splenic flexure artery [16].

The strengths with this study are the implementation of previously quality assessed research tools including 3D CT reconstructions and 3D printing that have been validated at surgery and have shown high specificity, sensitivity, accuracy and reliability [4, 45]. 3D Models were printed when it was deemed necessary in order to acquire better visuospatial understanding and tactile information.

Our research group has previously shown that our printed 3D models provide as accurate information as a CT virtual model and are beneficial due to the additional tactile information [46]. Knowledge acquired on vessel calibres, lengths, and positions of bifurcation from these 3D models, are ultimately used at surgery allowing the operating surgeon to correctly identify vascular structures and enabling him or her to perform a better and safer oncological surgery. Thus, the 3D model plays a role of the roadmap during surgery. The usefulness of 3D models is especially obvious in cases of intraoperative bleeding. In such cases knowledge of the individual patient anatomy derived from these 3D models allows and facilitates prompt management of the bleeding avoiding further damage caused by blindly suturing.

There are limitations with this study. The analysed cohort of patients represent a Norwegian population. Thus, the observed vascular anatomy may not necessarily reflect the anatomy in other ethnic groups.

In conclusion we state that the wide range of the possible MCA bifurcation positions that have been documented in this article should be taken into account when performing right or left colectomy since the position of the MCA bifurcation is crucial for the quality of lymphadenectomy. The aMCA occurs in 1 of 3 patients and its direct course to the left colonic flexure seems to indicate that when present this vessel needs to be taken into account when performing surgery for cancer of the splenic flexure.

We predict that 3D models, both vascular maps on paper and physical models, will be available as a routine for all colorectal operations in the future. Also, there will be virtual models with possible use of virtual reality googles peroperatively [47]. All these aids are enabling the surgeon to perform better surgery by being better aware of the anatomy.

## Supplementary information

Below is the link to the electronic supplementary material.

Supplementary material 1 (PDF 2194 kb). Supplementary 1: A virtual 3D PDF of mesenteric vascular anatomy of the patient with the replaced middle colic artery. In order to see the model, it is necessary to activate 3D content. The annotation is found by clicking “Detailed view” and “Annotation text”. MCA indicates the middle colic artery; MCV indicates the middle colic vein; IMA indicates the inferior mesenteric artery; IMV indicates the inferior mesenteric vein; ICA indicates the ileocolic artery; ICV indicates the ileocolic vein; JV indicates the jejunal vein

Electronic supplementary material 2 (MP4 3425 kb). Supplementary 2: A 3D virtual model of the mesenteric vascular anatomy in a patient with the accessory middle colic artery exported as a short video showing the close relationship between the accessory middle colic artery and the lower border of pancreas. For annotations, see Figure 5
